# Hotel Practice in New York

**Published:** 1854-05

**Authors:** 


### *Hotel Practice in New York—An Infernal Practice.*

"He was a stranger and we took him in."

The abuses of our profession demand the eyes of Argus, and the arms of Briareus. If father Jupiter paid that old coon for guarding Io no better than our brethren pay us for watching over their characters, we don't wonder Apollo has given so large a number of them to the devil. It would seem that "respectable gentlemen in black" (we think our brethren have selected a most appropriate color for their dress,) has given them special counsel in getting up the system of practice at present pursued in the "Hotel Practice" of our city. The cookery and ventilation in these "magnificent establishments," together with the refined and fastidious palates of a large portion of the traveling public, afford uncommon facilities for practice upon their bodies and their pockets. The physician who has given a philosophical glance at the valiant trenchermen engaged at their suppers on board a North River steamboat, and then, after fortifying his stomach with a glass of brandy-and-water, and his nose with a piece of camphor, descended into that "inferno," the lower cabin at midnight, has had a practical idea of the facilities for "Hotel Practice." On board the boat, the patient spends but one night; at the hotel, usually several; he is generally ready for practice by the third night, when the operation commences. Nine out of ten of the cases of sickness at these places are cholera morbus, demanding no more than a purgative, with a little laudanum, or tinct. Hyociamus, fresh air, and a little light soup; but getting considerably more, as you shall see. The modern discoveries in "Hotel Practice" may be of service to our country readers; if editors will give the hint, they will probably get no drinks gratis when they come to the city.

A violent pull at the bell summons the porter, who is requested to bring a doctor immediately; he may possibly be asked to bring a gentleman of character; 'tis all one, however, he has received his cue from the bar-keeper, between whom and the doctor there is "an arrangement." He assures the gentleman, in the midst of his writhings and groans, that Dr. Snooks is one of the first medical men in the city, whose skill has often been tested in the house; the Esculapian is summoned, and is soon at the bed-side. The sick man being in an admirable condition to acknowledge sympathy, receives it in abundance, and at suitable intervals a few calomel pills, and occasional reminders of the necessity of "doing something" at a lower portion of his intestinal tract. He is regaled at suitable intervals with a joke, a little laudanum, and peppermint or camphor, with a few drops from a wonderful little bottle, which the doctor takes from his side pocket; he is learnedly informed that the "primæ viæ must be cleared out." This is very satisfactory, and convinces him of the doctor's intelligence. The window is judiciously closed, for fear of his "taking cold." The doctor endures the poisoned atmosphere, which has mainly produced the attack, by the aid of an occasional escape and visit at the bar, or a drink from his pocket pistol, and a walk in the hall. Towards morning, if nature be merciful, and the pills be retained, relief follows. If the patient were now let alone, and could get a little fresh air, some clean and simple meat broth, and the attention of a mother, a wife, or a sister, he would be out next day; but this is no part of our philanthropist's plan; it wouldn't pay house-rent and horse-keep, and servants' hire. He is therefore well dosed for three days, to overcome "the tendency to inflammation of the bowels;" mustard plasters are liberally used, and he may thank heaven if he escapes leeching and blistering. When he evinces a disposition to bolt, and relates his former experience in a similar case, where he was not so fortanate as to meet with any one but his poor country doctor, (who, of course, knew nothing, and had only one old horse, and neither rent nor servants to pay,) he is frightened with tales of the "epidemic condition of the air in the production of dysentery, and several severe cases now under treatment." &c., &c., with the story of Mr. So-and-so, who "was doing very well till he insisted on going home, where he speedily died, &c., &c. Another week's treatment with tonics, is the consequence of this rascality, and a bill of $50 or $75, per centage to the bar-keeper off.

Those who come to the city with chronic diseases, desiring to submit to the treatment of some gentleman previously selected, generally escape this miserable rascality; by no means, however, without hints and inuendoes of the superior skill of their favorite physician, who may, however, never in his life have seen or treated such a case as the one at hand. There is not a practical man in this city, of any character, who is not perfectly aware of the truth of this expose, and we most earnestly hope this statement will be extensively copied.—Our editorial friends could not better serve the cause of humanity. More of this anon.—*N. Y. Scalpel.*

Good! The traveling public should see to this. Who does not know that the character of the profession is blackened by the conduct of certain charlatans, who by measures consistent with their principles, contrive to secure the patronage of Hotels, and the favor of proprietors, bar-heepers, maids, cooks, hostlers, and all connected with the house or domiciling in it? Woe! to that luckless wight, who *happens* to be called professionally there, the landlord looks doubtful and grum, the bar-keeper or clerk is loud in his praises of the "*house favorite*," the landlady lauds his extraordinary skill, the chamber-maid repeats the song, the porter consoles the patient with the assurance that his attending physician will *surely kill him*, one boarder never employs him in *any case*, another knows of a *number of instances* of his want of skill, and a third advises that he be *immediately discharged*, which, under the influence of this multiplied testimony, is immediately done, and the great, the learned, the triumphant is introduced after a silent exchange of a *chuckle* on the part of "mine host" and a complacent *grin* on the part of the favorite, which when interpreted would read "we have done them." This contemptible professional Iago, with two of the metalic requisites in his possession, namely, "silver in his tongue" and "brass in his face," proceeds to display *his* wisdom on the one hand, and expose the *ignorance* of the victim of this disgraceful plot on the other.

Now this system of management, for the patronage of a public house, is not only an "infernal abuse," to use the language of the caption to this article, but it is *corrupt and rotten.* In many instances, we have been informed, a gratuitous attention is given to the family in return for this patronage. The public should be aware of this practice, which is but too common, and when a hotel-keeper urges the claims of a *certain* medical man, suspicion should be awakened. We rejoice that this subject has been brought to the notice of the medical public by the "Scalpel," but it should not be confined to this range, it should have a wider one, the secular papers should take it up, that the sick traveler may not fall into the hands of a base, unprincipled deceiver, who cares less for his life than his money, for any one will admit that he who will condescend from the true standard of professional dignity and honor to such low tricks, will not hesitate to do any thing. Our country, city, and state societies should look to this matter and by a public *manifesto* correct the evil. Our merchants when they travel East should be made aware of these tricks and physicians should advise their friends when they leave home, to beware of falling into such traps. They are called upon to warn their friends against houses where the circumstances point to a *bargain* between proprietor and physician. A membership of the same church, or some other organization, are circumstances which often awakens a preference, although the recipient of such partiality is less entitled to patronage, mentally or morally, than others who scorn to be bought by such promises of favor. Ed.

